# Systematic literature review of the use of Staff Attitudes to Coercion Scale (SACS)

**DOI:** 10.3389/fpsyt.2023.1063276

**Published:** 2023-02-07

**Authors:** Tonje Lossius Husum, Johan Siqveland, Torleif Ruud, Jakub Lickiewicz

**Affiliations:** ^1^Faculty of Health Sciences, Oslo Metropolitan University, Oslo, Norway; ^2^Centre for Medical Ethics, University of Oslo, Oslo, Norway; ^3^Mental Health Services, Akershus University Hospital, Lørenskog, Norway; ^4^National Centre for Suicide Research and Prevention, Institute of Clinical Medicine, University of Oslo, Oslo, Norway; ^5^Institute of Clinical Medicine, University of Oslo, Oslo, Norway; ^6^Department of Health Psychology, Jagiellonian University Medical College, Kraków, Poland

**Keywords:** mental health, staff, attitudes, assessment, coercion

## Abstract

**Objective:**

Staff’s attitudes to the use of coercion may influence the number of coercive interventions employed and staff willingness to engage in professional development projects aimed at reducing the use of coercion itself. The Staff Attitude to Coercion Scale (SACS) was developed to assess the attitudes of mental healthcare staff to the use of coercion in 2008 and has been employed subsequently. This global study systematically reviews and summarizes the use of the scale in research.

**Methods:**

Seven databases were searched for studies using SACS in articles published in peer reviewed journals and gray literature. In addition, researchers who have asked for permission to use the scale since its development in 2008 were contacted and asked for their possible results. Extracting of data from the papers were performed in pairs of the authors.

**Results:**

Of the 82 identified publications, 26 papers with 5,838 respondents were selected for review. A review of the research questions used in the studies showed that the SACS questionnaire was mostly used in studies of interventions aimed at reducing coercion and further explain variation in the use of coercion.

**Conclusion:**

SACS is, to our best of knowledge, the only questionnaire measuring staff’s attitudes to the use of coercive interventions in mental health services. Its widespread use indicates that the questionnaire is perceived as feasible and useful as well as demonstrating the need for such a tool. However, further research is needed as the relationship between staff attitudes to coercion and the actual use of coercion remains unclear and needs to be further investigated. Staff attitudes to coercion may be a prerequisite for leaders and staff in mental healthcare to engage in service development and quality improvement projects.

## 1. Introduction

The use of coercive practices is contested, with a long history of debate and controversy within mental healthcare (MHC) services (1–3). In line with heightened focus on human and users’ rights in healthcare services in society in general, the health authorities in many countries and international organizations have advocated for a reduction in the use of coercive practices, developing and promoting services based on informed consent instead (4–6).

At the beginning of the 21st century (2003–2005), a multi-center study of the European Evaluation of Coercion in Psychiatry and Harmonization of Best Clinical Practice (EUNOMIA) was carried out to assess the extent of the use of coercion in psychiatry. The results showed differences in the frequency of the use of coercive measures not only between respective countries, but also between institutions or departments, while factors increasing the risk of coercion were divided into three independent groups: characterizing the patient, treatment center, and staff. The quality of work and the atmosphere in the ward, the relationship between patients and staff, and the experience of medical personnel are all important factors affecting the frequency of the use of coercion (7, 8).

As presented, previous research has shown considerable variation in the use of coercive measures in MHC within (9–11) and between countries (12–15). This variation indicates that some MHC institutions deliver care with less use of coercive measures than others (4, 6, 16), but factors influencing variation in the use of coercive practices are not fully understood. However, staff attitudes to the use of coercion are presumed to be one of many factors explaining this variation (17–19).

Attitudes are a central topic of study in social psychology, and a commonly accepted definition of attitudes are “a psychological tendency that is expressed by evaluating a particular entity with some degree of favor or disfavor” (20). Attitudes can vary along the dimensions of strength and valence, where strength indicates how strong the attitude is and valence refers to direction (either positive or negative). Examples of mechanisms forming attitudes are modeling, positive and negative reinforcement, and other learning mechanisms affecting a person’s evaluation of an object or phenomenon (21). Attitudes are often formed and changed in social interactions and through experience with the object of attitude (22). Individuals’ attitudes are assumed to influence their actual behavior. Research has, however, revealed that the connection between people’s attitudes and behavior are complex and less straightforward, as Ajzen (23) describes in “The theory of planned behavior” (23).

The “Staff Attitude to Coercion Scale” (SACS) was developed and published in 2008 as a valid and feasible instrument to measure staff attitudes to coercion (24). Since then, the questionnaire has been used in several countries (25–28). In addition to the original Norwegian and English translation, the SACS questionnaire has been translated into Farsi, German, Polish, and Chinese (25, 26, 28, 29). Japanese, Belgian, and Italian versions have been translated, but are not yet published.

In addition, the questionnaire has been employed in one study in populations with caregivers (30). The SACS questionnaire consists of 15 normative statements about the use of coercion, what one thinks about the use of coercion, and if one believes coercion should be used more or not. Normative statements contain a value judgment. It also contains statements that the use of coercion can cause various types of harm and offense to patients. The use of the questionnaire has shown that the items can be sorted into three dimensions:

SACS I: Coercion is needed for security and care reasons (pragmatic)

SACS II: Coercion may offend and harm patients (critical)

SACS III: Coercion is useful in treatment (positive) Psychometric properties have been assessed in a previous systematic review, which concluded that the included studies provided support for adequate structural validity and internal consistency, while other important measurement properties were not addressed by the studies reviewed. Caution is therefore warranted when interpreting the results of the SACS in terms of aspects such as reliability, criterion validity, and measurement error (31). This is, however, to our knowledge, the first attempt to perform a systematic literature review of the use and results of studies that have used the SACS questionnaire.

The review questions were:

1. How has the SACS questionnaire been used?
2. What research questions have answers been sought for using SACS?
3. What did studies using SACS find regarding staff attitudes to coercion?
  - Differences between professions.
    - Differences between genders.
    - Age group differences.
    - Work experience.
    - Relationship with other measures.
    - Can Staff Attitudes to Coercion be changed?
    -  Relationship between staff attitudes to coercion and actual use of coercion.

## 2. Methods

A systematic literature search was conducted by a librarian using the following databases: MEDLINE *via* EBSCOhost, PsycINFO *via* APA PsycNET, Embase *via* Elsevier, CINAHL *via* EBSCOhost, the Web of Science *via* Thomson Reuters, Google Scholar, and OpenGrey. The full search string can be found in Supplementary Appendix A. The search focused on identifying original published studies in the form of articles published in peer reviewed journals. PhDs were also included. In addition, researchers who have asked for permission to use the scale since its development in 2008 were contacted and asked for their possible results. Variants of the following terms were used as search terms:

• SACS
• Staff attitudes to the coercion scale
• Staff attitudes toward the coercion scale
• Staff normative attitudes toward coercion

**FIGURE 1:**
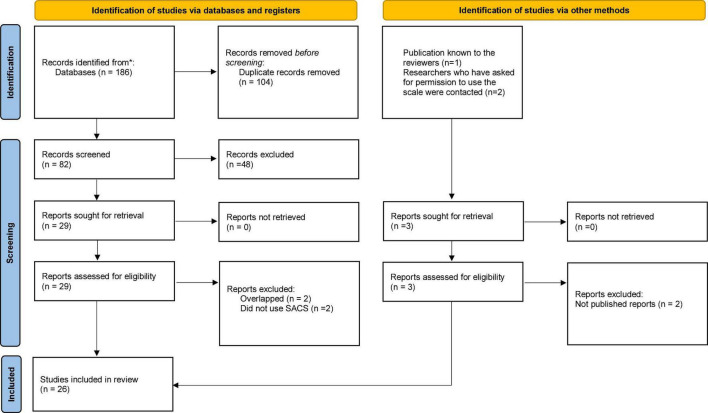
PRISMA 2020 flow diagram for new systematic reviews which included searches of databases, registers, and other sources [adapted from Page et al. (55) and Mahdanian et al. (6)]. *Number of records identified from each database is presented in the Supplementary Appendix A in the description of the database search.

No restrictions were placed on the language of publication. The first search was performed in February 2021, with an updated search being performed in October 2021. In the update of the search, six new publications were found, but the only one of these that met the inclusion criteria had already been included from other searches. After the initial assessment of the papers, 29 papers were read in full-text versions by two people in pairs, searching for eligibility. In the case of uncertainty, inclusion was discussed and agreed upon by a third reviewer. The systematic review was conducted according to PRISMA guidelines. Figure 1 shows a flow chart of the search process.

Extracting data from the papers were also performed in the same pairs of authors. Altogether, 26 papers were included in the review. A list of the 26 included papers is shown in Table 1.

**TABLE 1:** Information about included studies.

References	Country and language	Setting	Sample and size	Design	Research questions
Al-Maraira et al. (45)	Jordan/English	National Centre for Mental Health	48 Psychiatric nurses	Longitudinal/intervention	Evaluate the effectiveness of a training program on the psychiatric nurses’ attitudes
Al-Maraira and Hayajneh (44)	Jordan/English	National Centre for Mental Health	85 Psychiatric nurses	Cross sectional/correlation	Identify sociodemographic differences in staff attitudes to coercion
Arab et al. (25)	Iran/Farsi	Three hospitals	273 Staff (61% nurses, 11% physicians, 26% paramedics)	Psychometric/validation	Validate questionnaire and assess staff attitudes
Bartholomew (42)	USA, New Jersey/English	Three State Psychiatric Hospitals	232 Multiprofessional staff	Cross sectional/correlation, regression	Explore staffs’ exposure to, and attributions about responsibility for, violence, and attitudes to coercion
Efkemann et al. (26)	Germany/German	Two Psychiatric Hospitals	209 Mental health professionals (mostly nurses and doctors)	Psychometric/validation	Develop and validate German version of SACS
Elmer et al. (32)	Switzerland and Germany/ German	Inpatient psychiatric departments in five psychiatric clinics	424 Staff of inpatient psychiatric departments, 101 medical students at a Swiss university	Cross sectional/online survey	Professionals’ recognition of informal coercion and relationship to coercion
Gerace and Muir-Cochrane (47)	Australia/ English	Various types of services	512 nurses	Cross sectional/online survey	Investigate factors driving attitudes toward reducing and eliminating seclusion and restraint
Gowda et al. (46)	India/English	Department of Psychiatry	189 psychiatrists	Cross sectional/correlation	Assess clinicians’ attitude on the use of coercive measures
Hotzy et al. (33)	Switzerland/German	University hospital	107 MDs (43 psychiatrists, 64 physicians)	Cross sectional/correlation	Compliance of referring physicians comply with the legal requirements for involuntary admission
Husum (9)	Norway/ Norwegian	33 Psychiatric acute and subacute wards from all five health regions of Norway	651 Multiprofessional staff (74 MD, 21 psychologists, 335 nurses, 43 social workers, 78 enrolled nurses)	Cross sectional/correlation, multilevel analysis	Develop and validate the SACS questionnaire
Husum et al. (17)	Norway/ Norwegian	32 Psychiatric acute wards in Norway	529 Multiprofessional staff	Cross sectional/correlation, multilevel analysis	Investigate frequency and variance in the use coercion, predict use of coercion for involuntary admitted patients
Husum et al. (24)	Norway/ Norwegian	15 Acute and sub-acute psychiatric wards	215 Multidisciplinary staff members	Psychometric/validation	Measure factors influencing attitudes and differences between wards in staff attitudes to coercion
Jaeger et al. (35)	Switzerland/ German	Four acute psychiatric wards at one hospital	39 Multiprofessional staff	Cross sectional/correlation	Evaluate how staff recognize different levels of coercion and pressures and their attitude toward these interventions
Kiejna et al. (28)	Poland/Polish	Seven psychiatric wards	120 Multiprofessional staff	Psychometric/validation	Develop and validate Polish version of SACS
Krieger et al. (36)	Germany/ German	Psychiatric hospital	138 Multiprofessional staff	Cross sectional/correlation	Assess attitudes, reflective interventions and emotions toward coercion, and factors increasing the probability of coercion
Lambert et al. (37)	UK/English	Forensic mental health units Community mental health service	63 Nurses	Cross sectional/correlation	Explore how interpersonal style and professional boundaries relates to attitudes to coercion
Lickiewicz et al. (27)	Poland/Polish	Three provinces in Poland	361 (342 Nurses, 9 psychiatrists)	Psychometric/validation	Validate the SACS, compare attitudes between nurses and psychiatrists and the relationship to self-efficacy
Molewijk et al. (19)	Norway/ Norwegian	Seven wards in three mental health institutions	379 Multiprofessional staff	Cross sectional/correlation	Investigate professional and contextual characteristics and staff’s attitudes to coercion and moral doubt
Molewijk et al. (38)	Norway/ Norwegian	Same setting as above. Seven wards in three mental health institutions	Same sample as above 379 Multiprofessional staff	Longitudinal/intervention	Investigate how implementing “Ethics of care reflection groups” changed staff’s attitudes to coercion
Motteli et al. (39)	Switzerland/ German	Psychiatric hospital	110 Multiprofessional staff	Cross sectional/correlation	Examine individual and workplace characteristics and professionals’ attitudes to coercion
Orlick (43)	USA, Pennsylvania/ English	Urban inpatient psychiatric clinic	50 [32 Nurses, 18 Personal Care Technicians (PCT)]	Longitudinal/intervention	Investigate if implementing of “Enhanced de-escalation training” reduce use of restraints
Rabenschlag et al. (40)	Switzerland/ German	Admission ward	Intervention group: 17 Multiprofessional staff Control group = 21 staff	Longitudinal/intervention	Evaluate staff satisfaction, working circumstances, ward atmosphere, and attitudes toward recovery and coercion
Raveesh et al. (30)	India/English	Department of psychiatry	420 (210 psychiatrists, 210 caregivers)	Cross sectional/correlation	Compare the sociodemographic correlates of psychiatrists and caregivers’ attitudes to coercion
SINTEF Helse (41)	Norway/Norwegian	10 Acute psychiatric wards at six mental health hospitals	438 Multiprofessional staff (pre = 241; post = 197)	Longitudinal/intervention	Investigate if interventions reduce use of coercion
Vandamme et al. (18)	Germany/ German	13 Acute psychiatric wards at six hospitals	235 (93 Nurses, 56 psychiatrists)	Cross sectional/correlation, Randomized Controlled Trial (RTC)	Compare the predictive value of explicit (SACS) and implicit attitudes measures for the use of coercive measures
Wu et al. (29)	Taiwan/Chinese	Psychiatric hospitals and psychiatric wards in general hospitals	235 Psychiatric social workers	Cross sectional/correlation	Investigate values and attitudes toward involuntary hospitalization

## 3. Results

### 3.1. How has the SACS questionnaire been used?

Of the 26 included papers, 17 studies were conducted in Europe (17–19, 24, 26–28, 32–41), two in the USA (42, 43), three in the Middle East (Jordan and Iran) (25, 44, 45), two in India (30, 46), one in Australia (47), and one in Taiwan (29). Of these, eight studies had used an English translation (30, 37, 42–47), six the original Norwegian version (17, 19, 24, 34, 38, 41), eight a German translation (18, 26, 32, 33, 35, 36, 39, 40), two a Polish translation (27, 28), one in Farsi (25), and one in Chinese (29). Altogether, 5,838 informants participated in the studies, but some studies have probably included the same participants, so this number is just an estimate.

Most studies were descriptive and cross-sectional, while five studies were on interventions (38, 40, 41, 43, 45). One study used a pre-post measure design (41), and one study had a randomized controlled trial design (18). One study used a yes/no response instead of the original five-point Likert scale (30), and one study had used a four-point scale (36). Five of the studies had only translation and validation as its purpose (24–28). Two studies analyzed the single items, and not the three subscales (28, 47). Three studies confirmed the original three-factor model (25, 27, 28), and one study found that a one-factor model was better suited (26).

### 3.2. What research questions have been investigated using SACS?

The research questions in all the papers are shown in Table 2. A review of the research questions in the included studies showed that the SACS questionnaire was mostly used in studies aimed at reducing use of coercion or to try to explain variation in the use of coercive interventions. Some studies also aimed at explaining reasons for the use of coercive interventions and geographical variation in use. Five studies were solely validations and translations of the SACS in other cultural settings, and some aimed at describing staffs’ attitudes and investigating the differences between different staff groups. One study investigated why the use of coercion had not declined despite guidance from health authorities.

**TABLE 2:** (…) Means and standard deviations of the individual studies included in analysis.

References	Offending (M/SD)	Care and security (M/SD)	Treatment (M/SD)
Al-Maraira et al. (45)	N/A	N/A	N/A
Al-Maraira and Hayajneh (44)	N/A	N/A	N/A
Arab et al. (25)	3.58 (0.55)	3.71 (0.78)	3.05 (0.82)
Bartholomew (42)	3.24 (0.73)	3.56 (0.82)	2.53 (0.85)
Efkemann et al. (26)	3.30 (0.80) One dimention		
Elmer et al. (32)	3.55 (0.59)	4.15 (0.56)	1.93 (0.76)
Gerace and Muir-Cochrane (47)	N/A	N/A	N/A
Gowda et al. (46)	N/A	N/A	N/A
Hotzy et al. (33)	3.51 (0.60) Psychiatrists/ 3.19 (0.59) other physicians	4.26 (0.70) Psychiatrists/ 4.23 (0.61) other physicians	1.95 (0.78) Psychiatrists/ 2.27 (0.77) other physicians
Husum (9)	2.89 (0.56)	4.19 (0.49)	2.44 (0.68)
Husum et al. (17)	2.86 (0.24)	4.21 (1.6)	2.45 (0.21)
Husum et al. (24)	2.94 (0.95)	4.16 (0.72)	2.40 (0.94)
Jaeger et al. (35)	2.0 (0.4) One dimention		
Kiejna et al. (28)	N/A	N/A	N/A
Krieger et al. (36)	15.81 (3.44) Nurses, 15.89 (3.13) physicians 16.67 (3.51) psychologists 15.94 (3.60) male 15.88 (3.07) female	9.97 (3.23) Nurses, 8.96 (2.72) physicians 9.87 (3.04) psychologists 9.32 (3.19) male 9.58 (2.87) female	8.19 (1.92) Nurses, 9.14 (1.78) physicians 9.53 (1.76) psychologists 9.06 (1.93) male 8.49 (1.83) female
Lambert et al. (37)	17.95 (3.56) Min/max (6–30)	19.91 (4.93) Min/Max (6–30)	8.29 (2.75) (Min/Max 3–15)
Lickiewicz et al. (27)	2.75 (0.69)	4.25 (0.58)	2.44 (0.73)
Molewijk et al. (19)	3.13 (0.54)	4.12 (0.50)	2.58 (0.65)
Molewijk et al. (38)	3.11 (0.56) in T0 3.10 (0.55) in T1 3.13 (0.58) in T2	4.11 (0.50) in T0 4.01 (0.50) in T1 3.99 (0.55) in T2	2.58 (0.65) in T0 2.53 (0.68) in T1 2.50 (0.65) in T2
Motteli et al. (39)	3.67 (0.58) Open wards staff 3.33 (0.58) close wards staff	3.90 (0.75) Open wards staff 4.16 (0.43) close wards staff	1.68 (0.66) Open wards staff 1.94 (0.81) close wards staff
Orlick (43)	3.08 (1.09) Before training 3.24 (0.95) after training	3.36 (1.25) Before training 3.50 (1.25) after training	2.74 (1.20) Before training 2.64 (1.18) after training
Rabenschlag et al. (40)	1.99 (N/A) before and after intervention, 2.08 (N/A) control group		
Raveesh et al. (30)	3.9–5.1 (1.0–1.4) Age 4.2 (1.2) male 4.7 (1.2) female	4.7–5.1 (0.9–1.7) Age 4.9 (1.5) male 5.2 (0.8) female	1.0–1.1 (0.7–1.1) Age 1.0 (0.9) male 1.4 (0.9) female
SINTEF Helse (41)	3.2 (Mean of two measures)	4.4 (Mean of two measures)	2.3 (Mean of two measures)
Vandamme et al. (18)	2.2–2.63 (0.97–1.78) One dimention/in different clinics		
Wu et al. (29)	3.21 (N/A)	3.88 (N/A)	2.63 (N/A)

N/A = not available.

### 3.3. What did the studies using SACS find regarding staff attitudes to coercion?

Most staff considered the use of coercion as necessary for providing “care and security” and supported its use. On the whole, staff seemed to have a pragmatic view on the use of coercive interventions as needed for security reasons (29, 34) and that use of coercion was needed for security reasons: however, staff also commonly reported concerns about coercive measures potentially having offending and harmful effects on the patients (36, 47). Overall, the informants did not seem to view the use of coercive interventions as a treatment intervention (25). Two studies found that attitudes to coercion differed at an individual level between individuals, indicating that attitudes are influenced by individuals’ own personality and values (34, 41). Husum (9) found more difference between individuals than between staff groups (34). Further, two studies found significant differences between wards (34, 41), and one study found stable differences between wards across time (41). The same study found that staff on wards using the most coercive measures also had the most positive attitudes toward the use of coercive measures.

#### 3.3.1. Differences between professions

Of the studies with descriptive designs, many reported significant professional differences in attitudes (18, 36). One of the most consistent findings was that nurses were more positive toward coercive interventions and scored higher on the “Coercion as treatment” subscale than psychiatrists and psychologists (18, 27, 28, 36). One study found that most psychiatrists considered coercion as a caring, protective, and safety intervention, but also acknowledged its potential negative impact on patient dignity and therapeutic relationships (48). One study found that staff members with a university education scored lower on the “Coercion as offending” subscale compared to nurses (34); while one study found that staff with more education were significantly less in favor of use of coercion (43). This difference was especially visible concerning the “Coercion as treatment” subscale, where staff with undergraduate degrees were more likely to consider involuntary hospitalization as treatment compared to staff with a post-graduate degree, and to consider the use of coercive measures as treatment (29, 34). One study [Raveesh et al. (30)] compared attitudes to coercion between psychiatrists and caregivers. They found both psychiatrists and caregivers agreed that the use of coercion is related to scarce resources, security concerns, and harm reduction. Both groups agreed that coercion is necessary, but not as treatment. Both psychiatrists and caregivers considered coercion necessary for protection in dangerous situations. Interestingly, they found that reliability was reasonably good for psychiatrists, but not for caregivers (30).

#### 3.3.2. Gender differences

Three studies reported gender differences. Husum et al. (24) found that women had marginally lower scores on the “Coercion as treatment” subscale (24). Krieger et al. (36), however, found no significant gender differences on the subscales (36). Raveesh et al. (30) found that male psychiatrists considered coercion more related to scarce resources and violating patient integrity when compared to other staff (30).

#### 3.3.3. Age group differences

Husum et al. (24) found that staff older than 40 years considered use of coercion to be more an offense against patients than younger staff members (24). Krieger et al. (36), however, did not find any differences in attitudes related to age (36).

#### 3.3.4. Work experience

Four studies found that more work experience was related to a more critical view of coercion. Two studies found that staff members with the most work experience had a more critical view on “Coercion as offending” in comparison with less experienced staff (28, 36). Two studies found a positive correlation between working experience and the rating of “Coercion as care and security” (33, 44).

#### 3.3.5. Relationship with other measures

Nine studies assessed the relationship between SACS and other concepts and measures. Elmer et al. (32) found that the recognition and application of informal coercion correlated with attitudes toward coercion (32). Hotzy et al. (33) found that a higher score on the “Coercion as treatment” subscale correlated with staff finding it harder to comprehend the legal basis for decisions (33).

Jaeger et al. (35) found that the degree of coercion inherent to interventions comprising persuasion and leverage was underestimated by professionals with a positive attitude and overestimated by those with a negative attitude toward the respective interventions. They conclude that an advanced understanding of the influencing factors of professionals’ attitudes toward coercion could lead to improved training of professionals in utilizing interventions to enhance informed and ethical treatment strategies (35).

Lambert et al. (37) studied the relationship between SACS scores and the interpersonal style of staff. They found that the “Negotiator boundary management” style was associated with the “pragmatic attitude” to coercion, that is, a pragmatic view that coercion (and restrictive practices) is neither positive nor desirable but is sometimes necessary in order to maintain safety and security (37).

Molewijk et al. (19) investigated the relationship between staff being in “moral doubt” and the SACS scores. They found that staff who were more in doubt about the “moral rightness” of coercive interventions were in general more prone to possess a more critical view toward the use of coercion on the “Coercion as offending” subscale. Staff who were more occupied with morality were less prone to view coercion as treatment (19).

Two studies investigated the connection between SACS scores and recovery-oriented interventions. Molewijk et al. (49) studied the relationship between recovery expectations and attitudes to coercion. They found that the more optimistic recovery expectations staff possessed and working on open wards with fewer involuntary admissions and lower bed occupancy were associated with more critical attitudes to coercion (39). Rabenschlag et al. (40) used an RCT design to assess the implementation of a recovery-oriented ward concept and found that staff attitudes to coercion changed significantly in the intervention group compared to the control group (40).

Further, Vandamme et al. (18) studied the connection of staffs’ implicit and explicit attitudes toward use of coercion, and found no association (18). Lastly, Krieger et al. (36) investigated the relationship between staff attitudes to coercion and emotions, such as compassion, helplessness, grief, anxiety, sense of power, anger, guilt, and desperation, which seem to influence staff attitudes and suggest this as a future research topic (36).

#### 3.3.6. Can staff attitudes to coercion be changed?

Five studies report on change in staff attitudes to coercion. Al-Maraira et al. (45) found that staff attitudes showed change after implementing a training program aimed at altering attitudes based on Ajzen’s Theory of Planned Behavior. After four weeks of training, nurses in the intervention group demonstrated significant change in their attitudes’ mean scores, in the direction of gaining more critical attitudes toward use of coercive practices (45). Molewijk et al. (38) implemented the use of ethical reflection groups in staff groups and also found that attitudes (SACS scores) changed during time in a more negative/critical direction toward coercion after staff engaging in the ethical reflection groups (38).

Further, Orlick (43) found that, after implementing an intervention consisting of training in de-escalation techniques to reduce the use of restrains and seclusion, staff attitudes were found to be more critical to the use of coercion and more concerned about the potentially offending effects of coercive measures. There was, however, also a counterintuitive rise in the “security and care” scale (43). Rabenschlag et al. (40) investigated staff attitudes in relation with implementing a recovery-oriented ward concept and found that staff attitudes toward coercion did not change significantly in the intervention group but did so compared to the control group after intervention (recovery-oriented ward concept) (40). Last, an action-research project aimed at reducing the use of coercive interventions in hospitals found that staffs’ positive attitudes to coercion showed a slight decrease after the study period, as did the means for the actual use of coercion. Fewer staff scored neutral after the study period and showed stronger opinions about the statements in the SACS questionnaire. The findings were not significant, but there was a statistical tendency (41).

#### 3.3.7. Relationship between staff attitudes to coercion and the actual use of coercion

Five studies assessed the relationship between attitudes to coercion and the actual use of coercive interventions. Two of these found no substantial association (17, 18); one study found a tendency that staff on wards using the most coercive measures also had more positive attitudes toward coercive measures. The last study found that interventions (de-escalation techniques) did not reduce the use of restraints and seclusion, but staff attitudes did show some change after such interventions (43).

## 4. Discussion

Since its introduction in 2008, several research groups from many countries have used the SACS, which suggests that there is a need for such a tool and that it has been accepted as useful and feasible in the services.

The questionnaire has mainly been used in European countries but has also been used in India and in the Middle East (Jordan and Iran). Most of the studies confirmed the three-dimension model (with a possible exemption for the German translation). The German population was overall more critical to the use of coercion in care than other populations worldwide. One hypothesis could be that this reflects more positive attitudes toward patients in MHC in general in Germany.

On the whole, staff believe that the use of coercion is necessary for security reasons. We found more variation into what extent staff considered coercion could offend or harm patients. Further, we found that few staff considered the use of coercion as a treatment intervention. In general, nurses were found to be more in favor of coercive interventions than psychiatrists and psychologists. Less experienced staff were also found to be more in favor of the use of coercion. Further, we found studies that indicated that staff attitudes may be changed through targeted interventions such as ethical reflection groups and ethical training in general. When it comes to the connection between attitudes and the actual amount of use employed, the results were more divided.

The main reason for using the SACS scale has been in relation to professional development work to reduce the use of coercive interventions. This can be seen in relationship to the stronger emphasis to protect and promote users’ rights, which has been witnessed over the past 20 years (2, 4, 6). Since this review includes studies from the Middle East, India, and Taiwan, this seems to be a worldwide concern (25, 29, 45, 46). The scale has also been used to try to explain geographical variation in the use of coercive measures, which is a consistent finding in previous research (13, 14). Staff attitudes are often presumed to influence the use of coercive measures. What remains, however, is to establish the relationship between staff attitudes and the use of coercive interventions. Only one of the studies included in this review found a statistical tendency toward changes in staff attitudes being associated with a reduction in the use of coercion (41).

Several studies found, however, that staff attitudes could be altered through different kinds of professional development interventions. One of these interventions was the use of ethical reflection groups (49).

In sum, the overall findings about staff attitudes to coercion is that staff believe that the use of coercive measures are important tools in MHC to secure safety and to take care of people that need to be cared for. The findings indicate that staff are concerned with questions of safety. This indicates that, in order to change attitudes and reduce the use of coercive interventions, it is important to take staffs’ feelings, especially fear and perceived safety, seriously. Further, staff seem to not be very concerned about the potential harmful effects of coercive practices. Concerning the question about whether coercive practices are to be considered as treatment interventions, staff vary more in their views.

Descriptive studies of attitudes revealed differences between groups of professionals, mainly between psychiatrists and mental health nurses. Tendencies toward differences between staff with different amounts of experience and between men and women were also found. What remains, however, is to understand how attitude formation occurs. Attitudes may, for example, be developed through a socialization process during professional training itself (50).

Staff Attitude to Coercion Scale are often used in relation to attempts to reduce the use of coercive practices. Research on the relationship between attitudes and actual behavior shows, however, that it is complex (23). During the last 20 years, many attempts have been developed to reduce the use of coercive practices (2, 51). These are often developed as quality improvement projects in services and have showed positive results. In the compendium from WHO “Promoting person-centered and rights-based approaches in hospital-based mental health services,” some of these developments in successful service models promoting voluntariness in service delivery are presented (52). A summary of the attempts to reduce the use of coercive measures in mental health is that they are successful and reach their goals. However, staff attitudes to coercion may be a prerequisite for leaders and staff in MHC to engage in service development and quality improvement projects.

This systematic literature review has some practical implications. SACS can be used as a questionnaire for measuring the effectiveness of professional training. It might also be useful in assessing the risk of burnout. Further, staff members who perceived coercive measures as the best solution for the patient’s problematic behaviors may require support and additional training.

## 5. Conclusion and future directions

Staff Attitude to Coercion Scale is, to our best of knowledge, the only questionnaire measuring staff attitudes to the use of coercive interventions in MHC services. It is used widely, which demonstrates the need for such a tool. Its widespread use also indicates that the tool is perceived as feasible and useful. Most informants in the samples consider the use of coercion as necessary in potentially dangerous situations, although less agreement is found concerning the use of coercion as treatment and concern as to how the use of coercion may violate patients. The SACS is designated for MHC staff. In the future, it may also be developed to measure attitudes toward coercive intervention in MHC of other groups of informants, like the population in general, the police, or staff in primary healthcare. We found studies that indicated that staff attitudes may be changed through targeted interventions such as ethical reflection groups and ethical training. However, little is known about other types of training that might affect attitudes toward coercion. It would be interesting to see how other programs/models (like Safewards and Six-core strategy) could modify personnel attitudes (53, 54).

While not formally investigated in this review, changes in attitudes toward coercion in society at large may indicate that a revision of the SACS item may be warranted. After almost 15 years, some items seem outdated and some should possibly be altered. Examples of these are those items employing the words “regressive patients” and “insight.” Items may also be interpreted differently in different cultural settings. Topics for future research could therefore be to investigate cultural differences in staff attitudes to coercion more specifically. Another possible future direction in research on staff attitudes is to look more closely at staff emotions. Staffs’ feeling of safety may influence attitudes and the need to feel safe. Also needed is more investigation into the connection between staff attitudes to coercion and decision making in the process of using coercion. Another important topic for research is to investigate further how to motivate leaders in MHC services to engage in professional development work aimed at reducing the use of coercive practices, and especially how to use existing successful experiences and work to implement these in other services.

The SACS measures complex and sensitive issues, like using coercive measures. For this reason, some of the personnel members might modify their answers due to socially desirable response behavior. In future studies, it would be important to compare the SACS results and the social approval needs of the participants. A step in this direction could be to develop leadership and implementation skills in MHC staff, and to investigate the role of attitudes in implementation science. Most studies have used the three-factor model, but one study used a one-dimensional model. While the SACS is developed using three factors in staff attitudes, it could also be useful to explore the possibilities of developing a model that only uses a general attitude toward the use of coercion.

Analysis of the SACS means and standard deviation reveal, in some cases, different methods of counting the results. For this reason, differences found between the studies examined might be attributed not only to the different populations studied or the implementation of the study but also to the different methods of questionnaire validation. In the future, a systematic process of implementation across different countries and languages should include culturally- appropriate translations of the SACS.

### 5.1. Summary of findings

The SACS has mainly been used in European countries, India, and in the Middle East (Jordan and Iran).

The main reason for using the SACS scale has been in relation to professional development work to reduce the use of coercive interventions.

Most of the studies confirmed the three-dimension model (with one exemption for the German translation).

In general, staff believe that the use of coercive measures is important to secure safety and to take care of patients.

Further, staff seem to not be very concerned about the potential harmful effects of coercive practices.

Nurses were found to be more in favor of coercive interventions than psychiatrists and psychologists.

Less experienced staff were more in favor of the use of coercion.

Staff attitudes could be altered through different kinds of professional development interventions.

## 6. Limitations

The items in SACS are formulated in such a way that there is room for personal interpretations of them. The items, for example, do not address different kinds of coercive interventions like involuntary admission, coercive means, and involuntary medication. It is possible that informants view different kinds of coercive interventions differently; these kinds of differences cannot be distinguished using the questionnaire. A limitation with this systematic review is that not all studies have performed a quality insurance process of the translation. Used in different cultural settings, the items may also have different meanings. The scale has been translated and published into only a few languages (English, German, Polish, Farsi, and Chinese). Because of that, general conclusions regarding attitudes toward coercion are limited. It is difficult to compare and analyze cultural diversity. In the future, the availability of other languages should be promoted. Many of the studies are small professional development projects and should be interpreted with caution. A limitation with this study could be that two of the authors were also involved in the development of the original SACS questionnaire and could possibly be biased. We sought to take this into account by collaborating in pairs with the authors not included in the development of the questionnaire. All authors were also involved in the final interpretation of the findings. It is a challenge and possible limitation in this review that the validation process differs between different studies and countries. The SACS was developed and validated in 2008, but it is still popular and there might be a need to adapt the items to new environments and situations.

## Data availability statement

The raw data supporting the conclusions of this article will be made available by the authors, without undue reservation.

## Author contributions

All authors have been involved in the process of assessing the studies for inclusion, analyzing the results, and writing the manuscript.
